# A genomic search approach to identify esterases in *Propionibacterium freudenreichii *involved in the formation of flavour in Emmental cheese

**DOI:** 10.1186/1475-2859-7-16

**Published:** 2008-05-22

**Authors:** Julien Dherbécourt, Hélène Falentin, Stéphane Canaan, Anne Thierry

**Affiliations:** 1 INRA, Agrocampus Rennes, UMR1253 Science et Technologie du Lait et de l'Œuf, F-35000 Rennes, France; 2 CNRS, UPR 9025 Laboratoire d'Enzymologie Interfaciale et de Physiologie de la Lipolyse, F-13009 Marseille, France

## Abstract

**Background:**

Lipolysis is an important process of cheese ripening that contributes to the formation of flavour.* Propionibacterium freudenreichii *is the main agent of lipolysis in Emmental cheese; however, the enzymes involved produced by this species have not yet been identified. Lipolysis is performed by esterases (carboxylic ester hydrolases, EC 3.1.1.-) which are able to hydrolyse acylglycerols bearing short, medium and long chain fatty acids. The genome sequence of *P. freudenreichii *type strain CIP103027^T ^was recently obtained in our laboratory.

The aim of this study was to identify as exhaustively as possible the potential esterases in *P. freudenreichii *that could be involved in the hydrolysis of acylglycerols in Emmental cheese. The proteins identified were produced in a soluble and active form by heterologous expression in *Escherichia coli *for further study of their activity and specificity of hydrolysed substrates.

**Results:**

The approach chosen was a genomic search approach that combined and compared four methods based on automatic and manual searches of homology and motifs among *P. freudenreichii *CIP103027^T ^predicted proteins. Twenty-three putative esterases were identified in this step. Then a selection step permitted to focus the study on the 12 most probable esterases, according to the presence of the GXSXG motif of the α/β hydrolase fold family. The 12 corresponding coding sequences were cloned in expression vectors, containing soluble N-terminal fusion proteins. The best conditions to express each protein in a soluble form were found thanks to an expression screening, using an incomplete factorial experimental design. Eleven out of the 12 proteins were expressed in a soluble form in *E. coli *and six showed esterase activity on 1-naphthyl acetate and/or propionate, as demonstrated by a zymographic method.

**Conclusion:**

We were able to demonstrate that our genomic search approach was efficient to identify esterases from the genome of a *P. freudenreichii *strain, more exhaustively than classical approaches. This study highlights the interest in using the automatic search of motifs, with the manual search of homology to previously characterised enzymes as a complementary method. Only further characterisations would permit the identification of the esterases of *P. freudenreichii *involved in the lipolysis in Emmental cheese.

## Background

Lipolysis is an important process of cheese ripening that contributes to the formation of flavour in cheese. It occurs at various degrees according to the variety of cheese, and releases free fatty acids, which are involved in the characteristic flavour of cheeses [1,2]. The enzymes involved in lipolysis are milk lipoprotein lipase (LPL) and enzymes from cheese micro-organisms. New data were recently obtained regarding lipolysis in Emmental cheese. The concentrations of free fatty acids are about 4 mg/g of ripened cheese, and their profiles are roughly similar to that of fatty acids of milk triacylglycerols (TAG). About 15% of lipolysis occurs early during the first stages of Emmental cheese manufacture, and probably mainly results from the activity of LPL [3]. The remaining part of lipolysis takes place during ripening in the warm room, and is concomitant with the growth of *Propionibacterium freudenreichii*. This species is systematically used as a ripening culture in Emmental cheese, where it produces carbon dioxide, which results in the formation of the characteristic opening of this type of cheese [4]. *P. freudenreichii *is the main agent of Emmental cheese lipolysis [2,5], however the enzymes produced by this species that are involved in lipolysis have not yet been identified.

Lipolysis is performed by esterases (carboxylic ester hydrolases, EC 3.1.1.-), which are lipolytic hydrolases [6], commonly called lipases, and are able to hydrolyse insoluble acylglycerols at a water/lipid interface [7]. Some of the esterases implicated in the lipolysis in Emmental cheese are lipolytic hydrolases, able to hydrolyse acylglycerols present in Emmental cheese. Indeed, fat substrate of esterases in cheese is mainly TAG with various fatty acid chain lengths, i.e. from 4 to 18 carbon atoms [3], which constitute ~98% of the total lipids. TAG are originally present in bovine milk in fat globules constituted by a core of TAG surrounded by a biological membrane, which protects them from hydrolysis. When *P. freudenreichii *grows during ripening, however, the structure of fat is largely modified [8]. Fat globules are partially disrupted during milk transport and cheese manufacture, making TAG accessible to LPL until its inactivation by heating. The activity of *P. freudenreichii *enzymes on cheese fat could be facilitated by the fact that fat appears in Emmental cheese as large pockets. Moreover, colonies of bacteria are preferably localised in the fat/protein interface [3].

Several esterases have been described in *P. freudenreichii*, but only one CDS (CoDing Sequence) is ever known. An intracellular enzyme called 'lipase' was purified in *P. freudenreichii *by Oterholm *et al *in 1970 [9]. This enzyme was active on TAG bearing fatty acid chains of 4 to 8 carbons in length, but not on TAG bearing longer fatty acid chains. Therefore, the activity of this sole 'lipase' would hardly explain the release of fatty acids that is observed during cheese ripening. Most of the esterases characterised in *P. freudenreichii w*ere tested for their activity on soluble synthetic substrates such as phenyl-, and naphthyl esters [10,11], or on tributyrin [12]. This does not exclude that these characterised esterases could hydrolyse acylglycerols in Emmental cheese, but this hypothesis remains to be investigated. The only one known CDS of *P. freudenreichii *esterase, *estA*, was identified in *P. freudenreichii *JS strain after a random cloning, by sequencing a clone selected on tributyrin-agar plates [13]. This enzyme was not further characterised regarding its potential role in Emmental cheese lipolysis.

The aim of this study was to identify as exhaustively as possible the potential esterases in *P. freudenreichii *that could be involved in the hydrolysis of acylglycerols in Emmental cheese. The proteins identified were produced in a soluble and active form by heterologous expression in *Escherichia coli*. Their esterase activity was checked on 1-naphthyl propionate and acetate.

A genomic approach was chosen because it was considered as more powerful and exhaustive to identify esterases in *P. freudenreichii*, compared to the possible alternative strategies. For example, to tend to an exhaustive approach, it was considered inconceivable to perform a phenotypic screening after random cloning experiments in *E. coli *or mutagenesis in *P. freudenreichii*. Similarly, it appeared rash to purify the enzymes of interest from the small amount produced in *P. freudenreichii *cultures in which induction processes are not yet known. Moreover, the complete genome sequence of *P. freudenreichii *type strain CIP103027^T ^was recently completed in our laboratory. This genome sequence was automatically annotated using AGMIAL [14], and is currently being manually annotated. Up to date, the studies on esterases of sequenced organisms were mainly performed on predicted proteins that were annotated by homology searches with proteins from general databases. For example, the NCBI [15] "non-redundant" (nr) protein database is a general database commonly used for this kind of study as performed by Soror [16]. Putative esterases have also been identified by homology with previously characterised enzymes, for example by Ruiz [17]. Alternatively, consensus motifs have been designed from multiple sequence alignments especially around the active serine of different esterase families. The most represented family of esterases is the α/β hydrolase fold family that shows a minimal GXSXG motif around their catalytic serine. However, other families such as GDSL, GDXG, and patatin-like phospholipase with different motifs are well documented [18,19]. An additional conserved sequence, HG, partially constitutes the oxyanion hole in the three-dimensional structure of all lipolytic enzymes [20]. The search of motifs is currently mainly used either to classify enzymes identified by other methods such as homology searches, or to select the most probable active enzymes from a pool of annotated proteins [20]. We hypothesised that the search of motifs could also be useful to find putative enzymes. Searches of homology and motifs can be performed automatically by bioinformatics platforms, or manually with bioinformatics tools. Manual searches allow a more critical selection by the user.

In this study we developed a genomic search approach to identify as exhaustively as possible the putative esterases from the genome of *P. freudenreichii*, which could be involved in the formation of flavour of Emmental cheese by hydrolysing milk TAG with various chain lengths. This approach combined and compared four search methods based on automatic and manual searches of homology and motifs. The CDS of the 12 putative esterases identified were cloned and the best conditions to express the corresponding proteins in a soluble form determined. A soluble expression in *E. coli *of 11 out of the 12 proteins was performed in a large scale to assess their esterase activity by a zymographic method.

## Methods

### Bacterial strains, broths, and growth conditions

The *P. freudenreichii *and *E. coli *strains used in this study, as well as the plasmids and culture broths are listed in Table 1. *P. freudenreichii *was grown on Yeast Extract Lactate broth [21] at 30°C for 3 days. *E. coli *strains were grown on LB-agar (10 g/L) plates or in LB, SB or TB broths (see Table 1). When appropriate, antibiotics were added at the following concentrations: kanamycin, 50 μg/mL; ampicillin, 100 μg/mL; chloramphenicol, 30 μg/mL. For the duration and temperature of incubation, please refer to the cloning and screening section. *E. coli *growth was monitored by measuring the optical density at 600 nm (OD_600_).

**Table 1:** Strains, broths, plasmids, and primers used in this study.

Broths or strains or plasmids or primers	Relevant features	References or remarks
*P. freudenreichii*		
CIP103027^T^	*P. freudenreichii *sequenced type strain	Institut Pasteur^£^
		
*E. coli*		
DH10_B_	Host for pDONR221	Novagen^#^
B = BL21 (DE3) pLysS	Host for expression vectors	Novagen^#^
R = Rosetta (DE3)	Host for expression vectors	Novagen^#^
O = OrigamiB (DE3) pLysS	Host for expression vectors	Novagen^#^
C = C41 (DE3) pRos	Host for expression vectors	Avidis SA^$^
		
Broths		
LB	Miller's LB Broth Base	Invitrogen
SB	Superior Broth	Euromedex^§^
TB	Terrific Broth	Invitrogen
YEL	Yeast extract lactate broth	Malik *et al *[21]
		
Plasmids		
pDONR221	Gateway entry vector	Invitrogen
pETG20A	Expression vector	A. Geerlof*
pETG41A	Expression vector	A. Geerlof*
		
Primers^a^		
Forward	GGGGACAAGTTTGTACAAAAAAGCAGGCTCGGAAAACCTGTACTTCCAGGGTxx	This study
Reverse (attB2)	xxGGGGACCACTTTGTACAAGAAAGCTGGGTC	Invitrogen

^£^Institut Pasteur, Paris, France. ^#^Novagen, Gibbstown, NJ, USA. ^$^Avidis SA, Saint-Beauzire, France. ^§^Euromedex, Mundolsheim, France. *A. Geerlof, EMBL, Heidelberg, Germany.^a^xx indicates the start and the end of each *P. freudenreichii *sequence cloned.

### Genomic searches

The complete genome sequence of *P. freudenreichii *was mined using a prokaryote genome annotation system called AGMIAL [14], which was designed by INRA (Institut National de Recherche Agronomique, Jouy-en Josas, France). This annotation system predicts CDS (CoDing Sequences) and their corresponding proteins from the genome sequence. AGMIAL also executed requests using various tools. For example, BlastP [22] requests were used for protein homologies to automatically annotate the CDS. Furthermore, InterProScan [23] requests were used for motif searches to give elements to validate the automatic annotation. To identify putative esterases as exhaustively as possible, the predicted proteins were analysed by combining four methods. In method 1, searches were performed using AGMIAL for the name assigned by automatic annotation. This name corresponded to the most homologous protein found by BlastP with the NCBI nr protein database. In method 2, searches were performed using AGMIAL on the InterProScan motifs found. These first two methods were carried out by interrogating the result fields of AGMIAL requests. The searches included all the *P. freudenreichii *proteins whose automatic annotation or motif names contained the terms "esterase", "lipase", or "alpha beta hydrolase", e.g. esterase, lipase, but also phospholipase. In method 3, a manual search of homology was accomplished between all the proteins predicted by AGMIAL and 31 characterised lipolytic enzymes from PDB [24] and literature (data not shown). In method 4, the exact motifs related to carboxylic ester hydrolases and selected from Prosite (Table 2) were searched for in the predicted proteins using Fuzzpro from the emboss package [25]. Additionally, the minimal active serine motif (GXSXG) of the α/β hydrolase fold family and a part of the oxyanion hole sequence (HG) were searched for using Fuzzpro. The signal peptides were predicted using the SignalP 3.0 server [26].

**Table 2:** Motifs related to carboxylic ester hydrolases selected from Prosite.

Prosite pattern name	Accession number
Lipases, serine active site (GXSXG type)	PS00120
Lipolytic enzymes G-D-S-L family, serine active site	PS01098
Lipolytic enzymes G-D-X-G family, putative histidine active sites	PS01173
Lipolytic enzymes G-D-X-G family, putative serine active sites	PS01174
Carboxylesterases type-B1 (GXSXG type)	PS00122
Carboxylesterases type-B2	PS00941
Cutinase, serine active site (GXSXG type)	PS00155
Cutinase, aspartate and histidine active sites	PS00931
Pectinesterase signatures 1	PS00800
Pectinesterase signatures 2	PS00503

### Cloning of putative esterase CDS

The Gateway recombination system (Invitrogen, Carlsbad, CA, USA) was used to clone the putative esterase CDS of *P. freudenreichii *into pETG20A and pETG41A expression vectors. General molecular biology techniques were performed essentially as methods described previously by Sambrook *et al *[27]. The DNA of *P. freudenreichii *was extracted from an early stationary phase culture using a DNA extraction kit (Dneasy Tissue kit, Qiagen, Hilden, Germany). The DNA concentration was standardised to 50 ng/μL. Primers (Table 1) were designed to discard the ATG codon and to add the attB1 and attB2 recombination site at the 5' and 3' end, respectively. Moreover, the 5' primers coded for a 6 His-tag to facilitate the purification of the recombinant proteins and a Tobacco Etch Virus (TEV) Nia protease site to allow the fusion protein to be excised. The CDS were amplified by PCR in a 50 μL reaction volume using an Icycler thermocycler (Biorad, Hercules, CA, USA). Different DNA polymerases were used depending on the CDS and according to the manufacturers' instructions, with modifications. Six CDS, PF#169, PF#379, PF#667, PF#962, PF#1655, and PF#3004, were amplified (90 s at 94°C, 45 s at 64°C and 4 min at 72°C) 30 fold using 2.5 U Platinum Pfx DNA polymerase and 1× enhancer (Invitrogen). Three CDS, PF# 279, PF#774, and PF#1509, were amplified performing a 2 step reaction (40 s at 95°C and 4 min at 72°C) 30 fold using 2.5 U PicoMaxx HiFi PCR system (Stratagene, La Jolla, CA, USA). The three remaining CDS, PF#1637, PF#1861, and PF#2042, were also amplified performing a 2 step reaction (40 s at 98°C, and 4 min at 72°C) 30 fold using 1.25 U PrimeSTAR HS DNA Polymerase (TakaraBio, Inc., Otsu Shiga, Japan). PCR fragments were purified according to the manufacturer's instructions of the Gateway recombination system and resuspended in 20 μL of Tris EDTA buffer pH 8.0. They were then cloned into pDONR221, and then into the pETG20A and pETG41A expression vectors. The first cloning reaction was performed overnight at 25°C with 2 μL of pDONR221 at 150 ng/μL, 2 μL of BP Clonase II (BP clonase II enzyme mix (Invitrogen)), 8 μL of purified fragment, and 4 μL of Tris EDTA buffer. The second cloning reaction was performed overnight at 25°C with 2 μL of expression vector at 150 ng/μL, 1.5 μL of LR Clonase II (LR clonase II enzyme mix (Invitrogen)), 4 μL of purified recombinant pDONR221 at ~75 ng/μL, and 7.5 μL of Tris EDTA buffer. After each of these two cloning steps, recombinant plasmids were amplified in chemocompetent DH10B. Transformed cells were selected on LB-agar plates containing kanamycin for recombinant pDONR221 and ampicillin for recombinant expression vectors. The recombinant plasmids were amplified by growing a clone overnight in LB containing appropriate antibiotic, at 37°C under stirring at 200 rpm, and then purified using Nucleospin Multi-8-Plasmid kit (Macherey-Nagel, Hoerdt, France). Finally, the DNA sequences of each clone were confirmed by DNA sequencing (GATC Biotech AG, Konstanz, Germany).

### Screening for soluble expression

**Figure 1:**
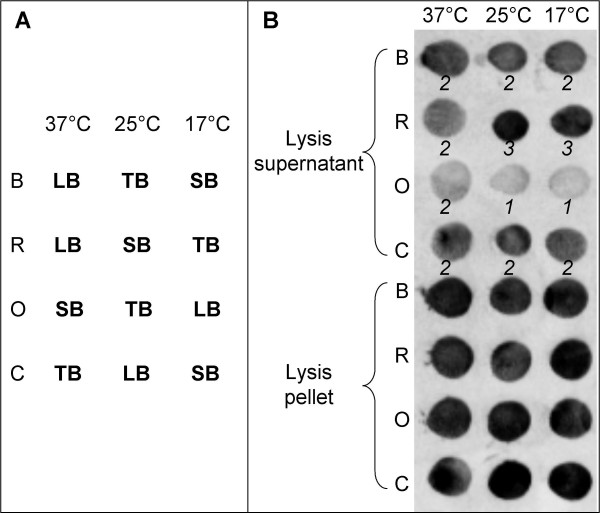
**Screening for recombinant protein expression**. (A) Schematic representation of the incomplete factorial experimental design showing the 12 tested combinations of three factors i.e. *E. coli *strain, expression temperature and culture broth (LB, SB, and TB). (B) Example of the dot blot from the expression screening of PF#1509 fused to the maltose binding protein (pETG41A). Numbers in italic are intensity scores from 0 to 3 attributed to each dot. The four strains of *E. coli *used for the screening were B (BL21 (DE3) pLysS), R (Rosetta (DE3)), O (OrigamiB (DE3) pLysS), and C (C41 (DE3) pRos).

The optimum conditions for soluble expression of each target were investigated using a factorial experimental design, previously described [28]. Briefly, the experimental design combined 4 *E. coli *strains, 3 expression temperatures (37°C, 25°C, and 17°C), and 3 culture broths (LB, SB, and TB), see Table 1. In accordance with Abergel's description, the factorial design was incomplete and designed by using the SAmBA software [29]. SAmBA computes the minimal number of experiments required for a solution to be found. Hence, only 12 combinations of factors were needed to be assessed out of the 36 possible. The 12 combinations tested are represented in Figure 1A.

Each expression vector was transformed into the four *E. coli *strains, selected on agar plates containing ampicillin and chloramphenicol. A single colony of each transformant was used to seed 4 mL of LB broth containing antibiotics in a 24 deep-well plate and pre-cultures were grown overnight at 37°C under stirring at 200 rpm. The following day, 4 mL of LB, SB, or TB broths containing antibiotics and distributed in three 24 deep-well plates, were seeded by 100 μL of overnight pre-cultures and incubated at 37°C under stirring at 200 rpm. When OD_600 _reached a value between 0.4 and 0.8, the expression of recombinant proteins was induced by the addition of 0.5 mM isopropyl β-D-1-thiogalactopyranoside. Expression was allowed to proceed under stirring at 200 rpm either for 3 h at 37°C, or overnight at 25°C, or overnight at 17°C. The OD_600 _of each culture was measured to calculate the volumes required to standardise cell numbers to a final OD_600 _equivalent of 10 in lysis solution. The different volumes of culture were transferred into 96 deep-well plates and spun at 2750 g for 20 min. The supernatant was discarded and the cell pellet re-suspended in 300 μL of lysis solution (50 mM Tris pH 8.0, 150 mM NaCl, 0.1% Triton X100, 1 mM EDTA, 0.25 mg/mL lysozyme) per well. The cell suspension was frozen for at least 30 min at -80°C. The suspension was thawed at 25°C and incubated for at least 30 min at room temperature under gentle stirring after the addition of MgSO_4 _and DNAse I to a final concentration of 20 mM and 10 μg/mL, respectively.

Due to the presence of a His-tag, the expression of recombinant proteins was assessed by a dot blot method using HRP conjugated anti-His antibodies and adapted from Vincentelli *et al *[30]. The suspensions of lysed cells were spun at 2750 g, 10°C for 45 min. Lysis supernatants were transferred into new 96 deep-well plates and the pellets were re-suspended in buffer A (50 mM Tris pH 8.0, 150 mM NaCl) containing 8 M urea, in order to solubilise insoluble proteins contained in the pellet. The proteins from supernatants and pellets were then purified by affinity chromatography for His-tag on a Protino Multi-96 Ni-IDA column (Macherey-Nagel). To purify the supernatant proteins, buffer A was used for column equilibration and washing steps. The elution step was performed using buffer A containing 500 mM imidazole. Identical buffers, however containing 8 M urea, were used to purify proteins contained in the pellet. One-hundred fifty microliters of each purified sample were loaded onto a polyvinylidene fluoride Hybond-P membrane (GE Healthcare Life Sciences, Piscataway, NJ, USA) using a vacuum manifold having 96 holes. Recombinant proteins were immunolabelled with Penta-His HRP Conjugate (Qiagen) diluted to 1/20,000 in blocking solution. Developing was performed with ECL Plus Western Blotting Detection Reagents (GE Healthcare Life Sciences) according to the manufacturers' recommendations. The intensity of expression was visualised by scanning membrane on a STORM imager (GE Healthcare Life Sciences). Intensity scores from 0 to 3 were attributed to each dot. The total score for each factor was calculated in order to determine the best conditions to express each recombinant protein in a soluble form.

### Protein identification and zymography

The 12 putative esterases were expressed in 100 mL culture by applying the best expression conditions found by the screening. Cells were disrupted as described above and the protein extracts were separated by SDS-PAGE in 12% polyacrylamide gels, according to Laemmli [31]. The molecular weight was appreciated using a low molecular weight marker (LMW Marker kit, GE Healthcare Life Sciences). The identity of each protein produced was checked by mass spectrometry after an in-gel trypsin digestion adapted from Shevchenko [32]. Briefly, gel pieces were excised from the gel, washed with acetonitrile and NH_4_HCO_3_, and then dried in a SpeedVac concentrator (SVC100H-200, Savant, Thermo Fisher Scientific, Waltham, MA, USA). In-gel trypsin digestion was performed overnight at 37°C and stopped with spectrophotometric grade trifluoroacetic acid (Sigma-Aldrich, St Louis, MO, USA). The supernatants containing peptides were then dried in a SpeedVac concentrator (Savant) and maintained at -20°C until mass spectrometry analysis. All mass spectra were obtained on a hybrid quadrupole time of flight (Q/TOF) mass spectrometer QStar XL (MDS Sciex, Toronto, Canada). In MALDI experiments, the samples were ionised with a laser beam (λ = 337 nm, 20 Hz) and peptide β-CN from β-casein (193–209) was used as a calibration standard. The more representative mono charged ions were submitted to fragmentation with collision energy comprised between 50 and 90 keV. Data-directed analysis was employed to perform MS/MS analysis on singly charged precursor ions. MS/MS spectra were collected from m/z 50 to m/z 2000. The mass spectrometer was operated in data-dependant mode automatically switching between MS and MS/MS acquisition using oMALDI Xpert 2.0 software (MDS Sciex). All data (MS and MS/MS) were then used with MASCOT (v.2.1) for search into a database containing all the predicted proteins of *P. freudenreichii *and Swissprot database to identify the proteins present in the sample. Depending on the quality of the spectra and/or score obtained, we considered as a prerequisite a minimum of at least two validated matching peptides for each protein investigated in the database search. Furthermore, the protein extracts were separated by native-PAGE in 10% polyacrylamide gels, i.e. a gel electrophoresis based on SDS-PAGE, but without SDS or any reducing agent. Esterase activity was assayed by zymography i.e. on activity gel according to Dupuis *et al *[12], with modifications. Briefly, native gels were rinsed in distilled water. Gels were then incubated for 1 h at 30°C in 0.1% Fast Red TR-salt (SERVA, Heidelberg, Germany) solutions that contained 2% of a 1% (w/v) 1-naphthyl propionate or 1-naphthyl acetate (Sigma-Aldrich) in acetone solution. Activities were visualised as brown bands.

## Results and Discussion

### Genomic searches

**Figure 2:**
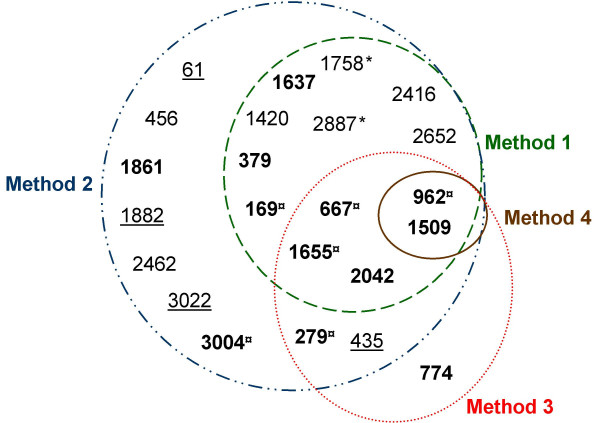
**Results of a genomic search approach using four methods and activity**. Twenty-three putative esterases were identified. **Method 1: **the results of the automatic search of homology used for automatic annotation by AGMIAL and containing the terms "esterase", "lipase", or "alpha beta hydrolase". **Method 2: **the results of the automatic search of motifs by InterProScan on the request of AGMIAL and containing the terms "esterase", "lipase", or "alpha beta hydrolase". **Method 3: **the results of the manual search of homology with 31 characterised lipolytic enzymes. **Method 4: **the results of the manual search of exact Prosite motifs related to carboxylic acid esterases. Twelve proteins (in bold) contained the GXSXG motif and were selected for the cloning of their CDS. ¤ pointed the selected proteins with esterase activity on 1-naphthyl acetate and/or 1-naphthyl propionate. Eleven proteins were not selected for the cloning of their CDS among which the four underlined proteins containing the GXSXG motif but corresponded to other α/β hydrolases and the two proteins indicated by an asterisk seeming to be predicted from pseudogenes belonging to a unique CDS truncated by a mutation (see text for details).

**Figure 3:**
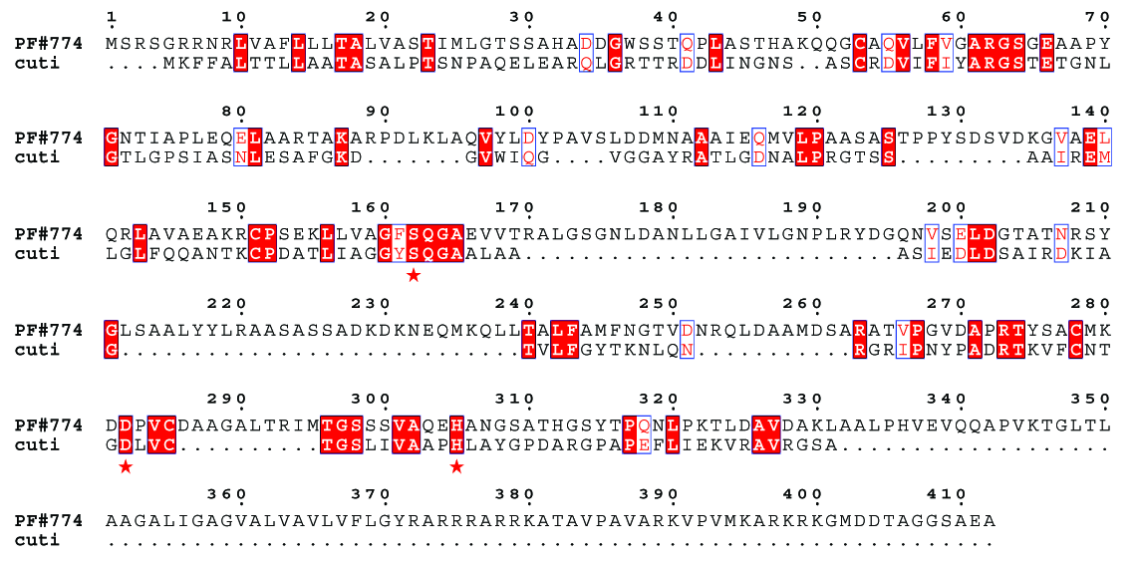
**Alignment between the cutinase of *Fusarium solani pisi *and PF#774 using BlastP**. Identical residues are indicated with red boxes and equivalent residues with white boxes. The 3 residues of the catalytic triad of *Fusarium solani pisi *cutinase (cuti) [PDB:1AGY] are indicated with asterisks.

The genomic search approach conducted in this study was composed of a search step which combined four methods to ensure that the greatest number of putative esterases were identified. Then, a selection step was necessary to focus the study on the most probable esterases, because some proteins identified, either did not have a catalytic serine, or corresponded to other well known enzymes. A schematic representation of these results is shown in Figure 2. Automatic interrogations of AGMIAL by method 1 for a protein homology search and method 2 for a motif search gave 13 and 22 positive results, respectively. Thirteen predicted proteins were commonly identified by both methods (Figure 2 and Table 3). Between these two search methods, the motif search appeared to be the most appropriate to find putative esterases since it yielded not only the same 13 found by homology but also 9 others. During searches of homology and motifs, the predicted proteins corresponding to enzymes capable of hydrolysing acylglycerols were retained, i.e. esterase, lipase, phospholipase, lysophospholipase, but also thioesterase (discussed hereafter). Indeed, all carboxylic ester hydrolases (EC 3.1.1.-) may possess a lipolytic activity and not only the subgroup of triacylglycerol lipase (EC 3.1.1.3). Moreover, the activity and the specificity of hydrolysed substrates of putative esterases cannot be inferred by bioinformatic analysis. Indeed, many factors may affect the enzyme/substrate complex, such as the secondary and tertiary structures. From these observations, a wide search for putative esterases was required to cover all possible esterase homology and motifs searches, regardless of the predicted activity. Thioesterases (EC 3.1.2.-), which act normally on thioester bonds, were also retained because some thioesterases have a hydrolase activity on carboxylic esters. For example, a thioesterase of *Mycobacterium smegmatis *hydrolyses monoolein [33], and thioesterase I of *E. coli *hydrolyses naphthyl, phenyl, benzyl, and p-nitrophenyl esters [34]. In contrast, Phosphoesterases were not retained in this study since they are phosphatases from the α/β hydrolase fold family. Despite their suggestive name they are more often associated with DNA polymerases as a catalytic domain [35]. Method 3, based on manual homology with 31 characterised lipolytic enzymes, allowed 8 putative esterases to be identified. Five out of these 8 predicted proteins were already identified by automatic search of homology (method 1) and 7 by automatic search of motifs (method 2). An additional protein, PF#774, was found by method 3. PF#774 had homology only with the lipolytic cutinase of *Fusarium solani pisi *[36,37] (Figure 3). This homology could be considered as weak, with an E Value of 0.23 (data not shown), a total homology of 23%, and 38% of positive residues. However, we considered it acceptable since it was strong around the serine active site. Thorough study has shown that a cutinase motif was found by InterProScan in PF#774. This putative esterase would also have been identified by automatic search of motifs if "cutinase" had been included as a search term. Regarding method 4 based on a search for all exact Prosite esterase motifs, it yielded only two positive results, PF#962 and PF#1509, with "Lipases, serine active site" motif. These two putative esterases were identified by the four methods used. By combining and comparing four search methods, the approach designed in this study highlighted the interest in searching for motifs with a tool such as InterProScan. Using appropriate terms for interrogating the results, including "cutinase", the 23 putative esterases could all be identified by method 2 which automatically searched for motifs using InterProScan. Thus, this study shows the importance of the terms used to identify the highest number of putative enzymes among the proteins predicted from a genome. For example, PF#169 would not have been detected without the use of the "alpha beta hydrolase" term. Homology searches (methods 1 and 3) appeared to be too restrictive. Indeed, searches of homology cannot be considered as exhaustive. Firstly, default parameters used for an automatic homology search are very stringent. These parameters could not have been changed in our study since they were applied by AGMIAL for homology searches in the whole genome in an annotation purpose. Secondly, manual searches of homology tend to identify only the enzymes that are similar to previously known enzymes. Moreover, homology searches run the risk of either matching with uncharacterised proteins from uncured databases, or matching with incorrectly annotated characterised enzymes. For example, the activities of most of the annotated esterases in the NCBI nr protein database have not been assayed. Only a further study of the specificity of hydrolysed substrates could permit the confirmation of the esterase activity of the identified putative esterases. Additionally, it is also interesting to note that *estA*, the sole esterase CDS previously identified in *P. freudenreichii*, was not identified by AGMIAL in the genome of *P. freudenreichii *CIP103027^T^. However, *estA *was cloned by a random method from *P. freudenreichii *JS strain [13]. *P. freudenreichii *CIP103027^T ^and JS strains seem to be genetically different regarding their esterase genes.

**Table 3:** Part of the data relating to the 23 putative esterases. [see Additional file 1] for complete data.

*P. freudenreichii *proteins predicted by AGMIAL	Method 1: Automatic annotation coming from the most homologous protein	Method 2: Exact nomenclature of InterProScan motifs containing the search terms	Predicted molecular weight (kDa)	EMBL accession number of CDS
PF#61	Proline iminopeptidase (PAP) (PIP) (Prolyl aminopeptidase)	esterase, est-lip-thio, a/b-hydrolase	39.0	[EMBL: AM944369]
**PF#169**	Acetyl esterase family enzyme	a/b-hydrolase	24.9	[EMBL: AM944370]
**PF#279**	no	esterase, est-lip-thio, a/b-hydrolase	46.4	[EMBL: AM944371]
**PF#379**	Putative phospholipase	patatin like, FabD/lysophospholipase	33.7	[EMBL: AM944372]
PF#435	Peptidase, S9C (Acylaminoacyl-peptidase) family (EC 3.4.-.-)	esterase, est-lip-thio, a/b-hydrolase	70.3	[EMBL: AM944373]
PF#456	Putative magnesium or manganese-dependent protein phosphatase	FabD/lysophospholipase-like	53.0	[EMBL: AM944374]
**PF#667**	Putative lysophospholipase (EC 3.1.1.5). 312 bp	esterase, est-lip-thio, a/b-hydrolase	34.2	[EMBL: AM944375]
**PF#774**	Hypothetical protein	no	42.9	[EMBL: AM944376]
**PF#962**	putative lysophospholipase	esterase, est-lip-thio, a/b-hydrolase	39.9	[EMBL: AM944377]
PF#1420	Patatin-like phospholipase	FabD/lysophospholipase	27.9	[EMBL: AM944378]
**PF#1509**	Alpha/beta hydrolase fold	esterase, est-lip-thio, a/b-hydrolase	29.6	[EMBL: AM944379]
**PF#1637**	Acyl-CoA thioesterase II (EC 3.1.2.-).	Acyl-coA-thioesterase	32.2	[EMBL: AM944380]
**PF#1655**	Putative esterase	esterase, est-lip-thio, a/b-hydrolase	29.5	[EMBL: AM944381]
PF#1758*	Putative esterase precursor	esterase, est-lip-thio, a/b-hydrolase	36.2	[EMBL: AM944382]
**PF#1861**	Helicase, C-terminal:Type III restriction enzyme	Phospholipase	116.7	[EMBL: AM944384]
PF#1882	Proline iminopeptidase	esterase, est-lip-thio, a/b-hydrolase	45.6	[EMBL: AM944385]
**PF#2042**	Acetyl esterase family enzyme	esterase, est-lip-thio, a/b-hydrolase	35.2	[EMBL: AM944386]
PF#2416	Patatin-like phospholipase	Patatin-like phospholipase	28.5	[EMBL: AM944387]
PF#2462	Polyphosphate kinase (EC 2.7.4.1)	Phospholipase	82.4	[EMBL: AM944388]
PF#2652	Thioesterase family protein	acyl-ACP-thioesterase/thioesterase	32.5	[EMBL: AM944389]
PF#2887*	Putative esterase precursor	a/b-hydrolase	14.4	[EMBL: AM944383]
**PF#3004**	(Acyl-carrier protein) S-malonyltransferase (EC 2.3.1.39)	FabD/lysophospholipase-like	33.0	[EMBL: AM944390]
PF#3022	Homoserine O-acetyltransferase (EC 2.3.1.31)	alpha/beta-Hydrolases	44.3	[EMBL: AM944391]

Twenty-three putative esterases were identified using four methods (see the legend of Figure 2). The results from method 1 and method 2, the predicted molecular weight, and the accession numbers of the CDS in EMBL database are specified for each of the 23 putative esterases identified. Twelve proteins (in bold) contain the GXSXG motif and were selected for the cloning of their CDS. Eleven proteins were not selected for the cloning of their CDS among which the four underlined proteins containing the GXSXG motif but corresponded to other α/β hydrolases and the two proteins indicated by an asterisk seeming to be predicted from pseudogenes belonging to a unique CDS truncated by a mutation (see text for details).

### Selection of 12 putative esterases for cloning

The searches of homology and motifs permitted to identify putative esterases only belonging to the α/β hydrolase family. From this observation, we selected as the most probable active esterases, the predicted proteins that contained the highly conserved GXSXG motif around the active serine, characteristic of the esterases belonging to the α/β hydrolase family. Seventeen predicted proteins met this criterion. Twelve predicted proteins were retained as putative esterases for the cloning of their CDS. The five remaining were not retained, although they contained a GXSXG motif [see Additional file 1], due to various reasons discussed hereafter. The 12 retained putative esterases also contained the HG sequence which partially constitutes the oxyanion hole. Eleven out of the 12 putative esterases contained exactly the GXSXG serine motif, and 1, PF#279, contained an inexact serine motif with a T instead of the first G. However, PF#279 had homologies with several characterised lipolytic enzymes, e.g. with the lactonizing lipase of *Pseudomonas aeruginosa*, particularly around the active serine motif and oxyanion hole sequence. Moreover, the TXSXG motif was also found in the LipB lipase of *Legionella pneumophila *[38]. The eventuality that the presence of the T could be due to a sequencing issue was discarded, because the percentage of coverage at this location was high. Moreover, the sequence was validated on the expression plasmids.

The five aforementioned predicted proteins, which possess the GXSXG motif but that were not retained for cloning, were PF#61, PF#435, PF#1758, PF#1882, and PF#3022. PF#1758 seemed to be predicted from a pseudogene as PF#2887 and both could belong to a unique CDS truncated by a mutation (data not shown). "Fragment of putative carboxylic ester hydrolase" was proposed as a new annotation for these two CDS. PF#61, PF#435, PF#1882, and PF#3022 belonged to other families of the α/β hydrolase fold family. The corresponding functions were proposed as new annotation for their four CDS, [see Additional file 1] for all new annotations. PF#1882 is a previously characterised proline iminopeptidase of *P. freudenreichii *[EMBL:AJ001361] [39] and PF#61 is a paralogous of PF#1882 in *P. freudenreichii *CIP103027^T^. PF#435 possess all the sequence characteristics of prolyl oligopeptidases (peptidase family S9) [40]. Finally, PF#3022 was not retained because it has strong homologies with the characterised homoserine O-transferases of *Rhodospirillum rubrum*, *Caulobacter crescentus*, and *Rhizobium loti *(data not shown), and contains a homoserine O-transferase motif [41].

Among the other non-retained CDS, PF#2462 was annotated as a "Polyphosphate kinase", according to its strong relating homologies (up to 59% of identity) and motifs with characterised polyphosphate kinases (data not shown). The remaining CDS were annotated as "Protein of unknown function" since they did not have any catalytic serine.

### Cloning of putative esterase CDS

In this study we chose to clone the CDS with hydrophilic fusion proteins and to screen the *E. coli *growth conditions to determine those which yielded the greatest soluble expression. Indeed, heterologous expression in *E. coli *often leads to insoluble proteins [42,43], especially in the case of lipolytic enzymes, due to their hydrophobic nature [44]. Consequently, cloning with hydrophilic fusion proteins and high throughput screenings have been developed to determine the experimental conditions that yielded either the greatest soluble expression [28,30], or the refolding of insoluble recombinant proteins [6] (for review see Sorensen et Mortensen 2005 [43]). Moreover, *P. freudenreichii *is a gram positive bacterium, with a high GC content (67%) [45], and is thus very different from *E. coli*.

The 12 CDS of the selected putative esterases were cloned into pETG20A and pETG41A expression vectors. These two vectors allow adding either soluble N-terminal thioredoxin (14.6 kDa) or maltose binding protein (43.3 kDa), respectively, to the proteins of interest. The pETG20A and pETG41A vectors were chosen because they give a better compromise between the length of fusion protein and the soluble amount of protein obtained after TEV cleavage of the fusion protein compared to other expression vectors such as pETG30A and pETG60A (A. Geerlof, EMBL) (Canaan, unpublished data). The cloning system allowed the cloning into both vectors of the CDS of the 12 selected putative esterases of *P. freudenreichii*. For 3 out of the 12 predicted proteins, namely PF#774, PF#1509, and PF#279A, a signal peptide was detected. Hence, their CDS were cloned without the DNA sequences corresponding to the signal peptides. The CDS were amplified by PCR using one out of three different DNA polymerases, depending on the CDS. Indeed, all the 12 DNA sequences could not be correctly amplified or amplified at all, using a single polymerase. The amplification issues that were met could be due to the high GC content of *P. freudenreichii*. Moreover, they were increased in this study by the use of very long primers (up to 78 nucleotides) to add the recombination sites, and which contained only 15 to 25 nucleotides specific to *P. freudenreichii *genes. The sequences of the cloned CDS were validated by sequencing in both vectors before further study.

### Screening for soluble expression

Soluble expression of the putative esterases was screened using a factorial design [28]. The expression of putative esterases fused to thioredoxin (pETG20A), the smallest fusion protein, was tested first. Even though all recombinant proteins fused to thioredoxin were expressed in a soluble form, the soluble amounts detected were small for PF#279 and PF#1509, and almost undetectable for PF#774, no matter the culture conditions and *E. coli *strain. Dot blots with *E. coli *lysis pellets showed these three recombinant proteins were effectively expressed, but mainly as inclusion bodies and in few conditions for PF#774. In this kind of screening, the results for the lysis pellets are expression control when a small level or no soluble expression was found for a recombinant protein. The expression of these three putative esterases, fused to the maltose binding protein (pETG41A), was thus tested. PF#279 and PF#1509 were expressed at greater soluble amounts with this second expression vector. An example of the dot blot from the expression screening of PF#1509 fused to the maltose binding protein is shown in Figure 1B. Regarding PF#774, the use of pETG41A did not improve the expression. No matter the expression vector used, *E. coli *growth was halted when the expression of PF#774 was induced, suggesting that this protein may be toxic for *E. coli*. This hypothesis could be verified by mutating the putative active serine by directed mutation of the PF#774 CDS in an expression vector.

Using the screening, we were able to find the best conditions to express in a soluble form 11 out of the 12 putative esterases retained. It should be noted that the best conditions differed between each protein, and between the two fusions of the same protein. For example, the best conditions to express PF#1509 fused either to thioredoxin, or to maltose binding protein (Figure 1B), were R (Rosetta (DE3)) grown in LB or TB at 37°C or 25°C, and R grown in SB at any one of the three temperatures, respectively. This demonstrates the interest in using a factorial experimental design. However, among the four *E. coli *strains tested in this study, R and C (C41 (DE3) pRos) globally gave the best results for the soluble expression of the putative esterases.

### Overexpression and esterase activity

Applying the combination of factors determined from the screening step, 11 proteins were successfully overexpressed in a soluble form on a larger scale (100 mL culture). When several strains of *E. coli *presented the same score in the screening step, each one was tested on a large scale. When several temperatures or broths presented the same score in the screening step, the lowest temperature and the less nutritive broth (LB<SB<TB) were chosen for large scale expression. The metabolism of *E. coli *is slower under these culture conditions, compared to higher temperature and more nutritive broth. Indeed, with slower metabolism, the expression rate is slower and may allow chaperonins to correctly fold the expressed protein, i.e. yielding soluble forms. For example, the expression screening of PF#1509 fused to maltose binding protein (Figure 1B) gave the same score at all temperatures, therefore 17°C was selected for protein expression.

**Figure 4:**
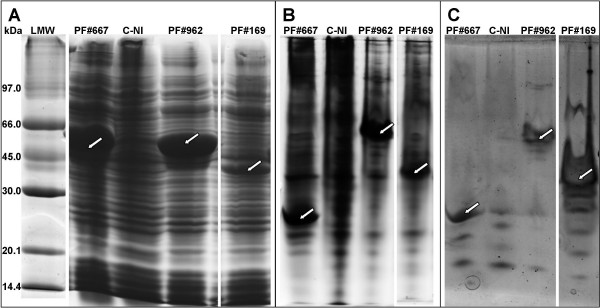
**Examples of PolyAcrylamide Gel Electrophoresis and activity on 1-naphthyl acetate**. Lysis supernatants of *E. coli *C41 (DE3) pRos, either expressing the recombinant proteins PF#667, PF#962, and PF#169 fused to the thioredoxin (14.6 kDa), or without induction of PF#962 expression (C-NI), were loaded onto polyacrylamide gels. (A) SDS-PAGE 12% stained with Coomassie Blue. LMW: low molecular weight marker (GE Healthcare Life Sciences). (B) Native-PAGE 10% stained with Coomassie Blue. (C) Activity on 1-naphthyl acetate revealed on native-PAGE 10% using Fast Red TR-salt. The arrows point the recombinant proteins of interest.

The experimental molecular weight of each recombinant protein, loaded onto 12% SDS-PAGE gels (Figure 4A), corresponded to the theoretical molecular weight. For example, molecular weights around 49 kDa, 55 kDa and 40 kDa were observed for PF#667, PF#962, and PF#169, respectively. These molecular weights corresponded to the predicted molecular weights (Table 3) added up to the 14.6 kDa of thioredoxin fusion protein. The identity of each protein produced was validated by mass spectrometry. Searches were performed into the predicted proteins of *P. freudenreichii *and also into the Swissprot database in order to avoid the validation of peptides from contaminant proteins.

For the 11 soluble proteins, the esterase activity was assayed directly from the lysis supernatants of *E. coli *expression cells, by zymography on 10% native-PAGE gels (Figure 4B). As *P. freudenreichii *possesses esterases hydrolysing 1-naphthyl acetate and 1-naphthyl propionate [12], zymography was performed using these two substrates. Zymography gave positive results, visualised as brown bands of high intensity, for 6 out of the 11 samples tested (Figure 4C). PF#169, PF#667, PF#962, PF#1655, and PF#3004 were active on both substrates and PF#279 was only active on 1-naphthyl propionate. "Carboxylic ester hydrolase" was proposed as the new annotation for the CDS of these six enzymes, whose esterase activity was biochemically proven. "Putative carboxylic ester hydrolase" was proposed for the CDS of the six remaining putative esterases. Some activity bands of low intensity were observed in all lines, including the control line (C-NI) with lysis supernatants of *E. coli *C41 (DE3) pRos without induction of PF#962 expression (Figure 4C). This suggests that these activity bands corresponded to esterases of *E. coli*. Neither insoluble substrate nor acylglycerols of Emmental cheese fat were tested in this study, since further purification and cleavage of the fusion protein would be necessary to show activity on this kind of substrate. Indeed, the intracellular components of *E. coli*, the lysis solution, and the fusion protein could prevent the enzymes to act at the interface formed with insoluble substrates and conceal their enzyme activities.

Esterase activity was observed for enzymes resulting from the four search methods of putative esterase. The esterase activity shown for PF#3004, which was identified only with method 2 (Figure 2), reinforced the interest shown in using automatic search of motifs to identify new esterases. Interestingly, an esterase activity was shown for PF#279, which possesses a TXSXG motif instead of the consensus GXSXG motif. This highlights the importance of the manual search of homology (method 3). Method 3 cannot identify all putative esterases, when compared to the powerful automatic search of motifs (method 2), but could be considered as complementary to method 2. Indeed, method 3 can allow putative esterases with motifs diverging from the consensus to be identified and prevent true carboxylic ester hydrolases to be discarded during the selection step. Moreover, when a new enzyme has an uncommon motif, no consensus can be made and only homology searches can allow new enzymes of this new family to be found.

## Conclusion

In this study, we were able to demonstrate that our genomic search approach was efficient to identify the putative enzymes among the proteins predicted from the genome of *P. freudenreichii*, more exhaustively than classical approaches. This study also highlighted the requirement of using a method based on the automatic search of motifs, with bioinformatic tools such as InterProScan, to be exhaustive. Using appropriate terms for interrogating the results, the automatic search of motifs can yield not only the same 10 putative esterases selected for the cloning of their CDS by combining the two mainly performed methods based on homology, but also two others. Moreover, one out of the two putative esterases exclusively identified by automatic search of motifs actually had an esterase activity. The automatic search of motifs is a powerful method, whilst keeping in mind the importance of the selection of the terms used to interrogate the results. The three other methods tested in this study were effective in identifying esterases, but were less powerful. They confirmed a part of the results found by automatic search of motifs. Moreover, the manual search of homology with characterised enzymes is complementary to the automatic search of motifs. This method could permit either to identify putative enzymes with unusual motifs, or homologous to particular enzymes such as cutinases, or to prevent inappropriate discarding during a selection step.

Our genomic search approach permitted us to identify 23 putative esterases from the genome of *P. freudenreichii *CIP103027^T^. After a selection step based on the presence of the GXSXG motif of the α/β hydrolase fold family, 12 putative esterases were retained as more probable esterases and cloned in *E. coli*. The cloning and screening strategies used led to a successful expression in a soluble form on a large scale of 11 of these 12 selected putative esterases. The remaining putative esterase was not produced since it appeared to be toxic for *E. coli*. A zymographic test, preliminary to a further characterisation, showed an esterase activity on short chain naphthyl esters for 6 out of the 12 expressed putative esterases. Thus, these esterase activities confirmed the efficiency of our genomic search approach.

The six active esterases identified as well as the six putative remaining are potentially able to hydrolyse acylglycerols of Emmental cheese. Only further study could permit to validate this hypothesis. After purification and cleavage of the fusion protein, each of the 11 proteins produced could be further characterised using substrates like monoacylglycerols, diacylglycerols, and TAG with various fatty acid chain lengths and milk fat. Moreover, knowing the CDS of the enzymes identified, the expression of their genes could be monitored by methods such as RT-PCR. The results of these further studies would permit to identify the esterases of *P. freudenreichii *involved in the lipolysis, thus in the formation of flavour in Emmental cheese.

Finally, ester synthesis also plays a role in the formation of flavour in cheeses [46] and is also performed by esterases. However, the direct involvement of *P. freudenreichii *enzymes in ester synthesis has not yet been demonstrated. The putative esterases of *P. freudenreichii *produced in our study could be tested for ester synthesis.

## Competing interests

The authors declare that they have no competing interests.

## Authors' contributions

JD participated in the design of the study, carried out the genomic searches and all the experiments (cloning, expression and characterization of proteins), and drafted the manuscript. HF participated in the genomic searches and the cloning. SC conceived of the cloning and expression studies and participated in the analysis and interpretation of data and critically revising the manuscript. AT conceived of the study, participated in its coordination, and helped to draft the manuscript. All authors read and approved the final manuscript.

## Supplementary Material

Additional file 1 Detailed results and data relating to the 23 putative esterases (rest of Table 3). The data provided represent the Table 3 supplemented by the GXSXG motifs and the HG sequence found, and the new annotation proposed. Click here for file
