# Supplementary material for: A genomic search approach to identify esterases in Propionibacterium freudenreichii involved in the formation of flavour in Emmental cheese
Source: Microb Cell Fact. 2008 May 22;7:16. doi: 10.1186/1475-2859-7-16 (PMC2442053; doi:10.1186/1475-2859-7-16)
Supplement: Additional file 1 — Detailed results and data relating to the 23 putative esterases (rest of Table 3). The data provided represent the Table 3 supplemented by the GXSXG motifs and the HG sequence found, and the new annotation proposed. [file 1475-2859-7-16-S1.doc]

| *P. freudenreichii* proteins predicted by AGMIAL | Method 1: Automatic annotation coming from the most homologous protein | Method 2: Exact nomenclature of InterProScan motifs containing the search terms | GxSxG motif | HG sequence | Predicted molecular weight (kDa) | New annotation proposed | EMBL accession number of CDS |
| --- | --- | --- | --- | --- | --- | --- | --- |
| PF#61 | Proline iminopeptidase (PAP) (PIP) (Prolyl aminopeptidase) | esterase, est-lip-thio, a/b-hydrolase | GGSWG | HGG | 39.0 | Proline iminopeptidase | [EMBL: AM944369] |
| **PF#169** | Acetyl esterase family enzyme | a/b-hydrolase | GFSAG | HG | 24.9 | Carboxylic ester hydrolase | [EMBL: AM944370] |
| **PF#279** | no | esterase, est-lip-thio, a/b-hydrolase | **T**HSMG | HG | 46.4 | Carboxylic ester hydrolase | [EMBL: AM944371] |
| **PF#379** | Putative phospholipase | patatin like, FabD/lysophospholipase | GISAG | HG | 33.7 | Putative carboxylic ester hydrolase | [EMBL: AM944372] |
| PF#435 | Peptidase, S9C (Acylaminoacyl-peptidase) family (EC 3.4.-.-) | esterase, est-lip-thio, a/b-hydrolase | GGSWG/ GGSAG | HGG | 70.3 | Peptidase, S9C (Acylaminoacyl-peptidase) family | [EMBL: AM944373] |
| PF#456 | Putative magnesium or manganese-dependent protein phosphatase | FabD/lysophospholipase-like | no | HG | 53.0 | Protein of unknown function | [EMBL: AM944374] |
| **PF#667** | Putative lysophospholipase (EC 3.1.1.5) | esterase, est-lip-thio, a/b-hydrolase | GHSWG | HG | 34.2 | Carboxylic ester hydrolase | [EMBL: AM944375] |
| **PF#774** | Hypothetical protein | no | GFSQG | HG | 42.9 | Putative carboxylic ester hydrolase | [EMBL: AM944376] |
| **PF#962** | putative lysophospholipase | esterase, est-lip-thio, a/b-hydrolase | GHSTG | HG | 39.9 | Carboxylic ester hydrolase | [EMBL: AM944377] |
| PF#1420 | Patatin-like phospholipase | FabD/lysophospholipase | no | no | 27.9 | Protein of unknown function | [EMBL: AM944378] |
| **PF#1509** | Alpha/beta hydrolase fold | esterase, est-lip-thio, a/b-hydrolase | GHSMG | HG | 29.6 | Putative carboxylic ester hydrolase | [EMBL: AM944379] |
| **PF#1637** | Acyl-CoA thioesterase II (EC 3.1.2.-). | Acyl-coA-thioesterase | GLSMG | HG | 32.2 | Putative carboxylic ester hydrolase | [EMBL: AM944380] |
| **PF#1655** | Putative esterase | esterase, est-lip-thio, a/b-hydrolase | GSSAG | HGGG | 29.5 | Carboxylic ester hydrolase | [EMBL: AM944381] |
| PF#1758* | Putative esterase precursor | esterase, est-lip-thio, a/b-hydrolase | GYSQG | no | 36.2 | Fragment of putative carboxylic ester hydrolase | [EMBL: AM944382] |
| **PF#1861** | Helicase, C-terminal:Type III restriction enzyme, res subunit: DEAD/DEAH box helicase, N-terminal | Phospholipase | GSSSG | HG | 116.7 | Putative carboxylic ester hydrolase | [EMBL: AM944384] |
| PF#1882 | Proline iminopeptidase | esterase, est-lip-thio, a/b-hydrolase | GQSFG/ GASGG | HG | 45.6 | Proline iminopeptidase | [EMBL: AM944385] |
| **PF#2042** | Acetyl esterase family enzyme | esterase, est-lip-thio, a/b-hydrolase | GFSAG | HG | 35.2 | Putative carboxylic ester hydrolase | [EMBL: AM944386] |
| PF#2416 | Patatin-like phospholipase | Patatin-like phospholipase | no | HGG | 28.5 | Protein of unknown function | [EMBL: AM944387] |
| PF#2462 | Polyphosphate kinase (EC 2.7.4.1) | Phospholipase | no | no | 82.4 | Polyphosphate kinase | [EMBL: AM944388] |
| PF#2652 | Thioesterase family protein | acyl-ACP-thioesterase/ thioesterase | no | HG | 32.5 | Protein of unknown function | [EMBL: AM944389] |
| PF#2887* | Putative esterase precursor | a/b-hydrolase | no | HG | 14.4 | Fragment of putative carboxylic ester hydrolase | [EMBL: AM944383] |
| **PF#3004** | (Acyl-carrier protein) S-malonyltransferase  (EC 2.3.1.39) | FabD/lysophospholipase-like | GHSVG | HG | 33.0 | Carboxylic ester hydrolase | [EMBL: AM944390] |
| PF#3022 | Homoserine O-acetyltransferase (EC 2.3.1.31) | alpha/beta-Hydrolases | GGSLG | no | 44.3 | Homoserine-O-acetyltransferase | [EMBL: AM944391] |

**Detailed results and data relating to the 23 putative esterases (rest of Table 3)** Twenty-three putative esterases were identified using four methods (see the legend of Figure 1). The results from method 1 and method 2, the exact GXSXG motifs, the HG sequence, the predicted molecular weight, the new annotation proposed in this study, and the accession numbers of the CDS in EMBL database are specified for each of the 23 putative esterases identified. Twelve proteins (in bold) contain the GXSXG motif and were selected for the cloning of their CDS. Eleven proteins were not selected for the cloning of their CDS among which the four underlined proteins containing the GXSXG motif but corresponded to other α/β hydrolases and the two proteins indicated by an asterisk seeming to be predicted from pseudogenes belonging to a unique CDS truncated by a mutation (see text for details).
